# A First in Human Trial Implanting Microalgae Shows Safety of Photosynthetic Therapy for the Effective Treatment of Full Thickness Skin Wounds

**DOI:** 10.3389/fmed.2021.772324

**Published:** 2021-11-30

**Authors:** Miguel Luis Obaíd, Juan Pablo Camacho, Marianne Brenet, Rocío Corrales-Orovio, Felipe Carvajal, Ximena Martorell, Consuelo Werner, Valeska Simón, Juan Varas, Wilfredo Calderón, Christian Dani Guzmán, María Rosa Bono, Sebastián San Martín, Antonio Eblen-Zajjur, José Tomás Egaña

**Affiliations:** ^1^Department of Plastic Surgery, Hospital del Salvador, Santiago, Chile; ^2^Institute for Biological and Medical Engineering, Faculty of Engineering, Pontifical Catholic University of Chile, Santiago, Chile; ^3^Division of Hand, Plastic and Aesthetic Surgery, University Hospital Ludwig Maximilian University of Munich, Munich, Germany; ^4^Critical Patient Unit, Hospital del Salvador, Santiago, Chile; ^5^Department of Nursing, Clínica Alemana, Santiago, Chile; ^6^Department of Biology, Faculty Science, Universidad de Chile, Santiago, Chile; ^7^Biomedical Research Center, School of Medicine, Universidad de Valparaíso, Valparaíso, Chile; ^8^Faculty of Medicine, School of Medicine, Universidad de Chile, Santiago, Chile; ^9^Sky-Walkers SpA, Litueche, Chile; ^10^Translational Neuroscience Lab, Faculty of Medicine, Universidad Diego Portales, Santiago, Chile

**Keywords:** human photosynthesis, clinical trial, skin regeneration, microalgae, photosynthetic biomaterial, skin wounds, photosynthetic therapy

## Abstract

Insufficient oxygen supply represents a relevant issue in several fields of human physiology and medicine. It has been suggested that the implantation of photosynthetic cells can provide oxygen to tissues in the absence of a vascular supply. This approach has been demonstrated to be successful in several *in vitro* and *in vivo* models; however, no data is available about their safety in human patients. Here, an early phase-1 clinical trial (ClinicalTrials.gov identifier: NCT03960164, https://clinicaltrials.gov/ct2/show/NCT03960164) is presented to evaluate the safety and feasibility of implanting photosynthetic scaffolds for dermal regeneration in eight patients with full-thickness skin wounds. Overall, this trial shows that the presence of the photosynthetic microalgae *Chlamydomonas reinhardtii* in the implanted scaffolds did not trigger any deleterious local or systemic immune responses in a 90 days follow-up, allowing full tissue regeneration in humans. The results presented here represent the first attempt to treat patients with photosynthetic cells, supporting the translation of photosynthetic therapies into clinics.

**Clinical Trial Registration:** www.clinicaltrials.gov/ct2/show/NCT03960164, identifier: NCT03960164.

## Introduction

Oxygen, the key molecule for aerobic metabolism, is fundamental to several cellular processes including mitochondrial respiration and reactive oxygen species production. Oxygen is also essential to nearly every step of the wound healing process, such as cell proliferation, collagen synthesis, angiogenesis and bacterial defense (1). Thus, hypoxia is broadly described as the leading cause for chronic wounds. Traditional systemic oxygen therapies, such as supplemental oxygen or hyperbaric oxygen therapy provide only modest support of wound healing (2, 3). To improve local delivery of oxygen to the wound site, oxygen-releasing dressings and topical oxygen therapy (4) have been extensively studied; however, inconsistent clinical data have thus far limited translation of these technologies (5, 6).

Given that oxygen is produced by photosynthetic organisms such as plants and cyanobacteria, the use of photosynthetic cells represents an attractive alternative for local oxygen delivery *in vitro* and *in vivo*. For instance, it has been shown that the presence of photosynthetic cells allowed the development of thicker three-dimensional cardiac structures *in vitro* (7), and improved cardiac function after heart ischemia *in vivo* (8). Additionally, photosynthetic cells sensitize cancer cells to radiotherapy and photodynamic therapies by increasing oxygen tension in hypoxic tumors *in vitro* and *in vivo* (9–12). In the context of wound healing, we have shown that photosynthetic biomaterials could contribute to tissue regeneration by increasing the local oxygen tension in wounds, independent of blood supply. First, the concept of photosynthetic biomaterials was introduced, loading commercially available scaffolds with microalgae, generating oxygen upon light stimulation *in vitro* (13). This approach was then validated *in vivo* in full thickness animal skin defects, demonstrating its safety and proposing that such oxygen-producing biomaterials could be a promising platform toward autotrophic engineered tissues (14). In addition to targeted oxygen delivery, photosynthetic microalgae engineered to release recombinant growth factors *in situ* demonstrated novel utility as vehicles for gene therapies in tissue regeneration *in vivo* (15, 16). These developments led to the generation of photosynthetic sutures for local and controlled delivery of oxygen and recombinant growth factors in wounds (17). Additionally, as recently demonstrated by an independent group, photosynthetic biomaterials have shown to promote wound healing in diabetic mice (18).

The use of photosynthetic cells for the local delivery of oxygen is a promising approach and, consequently, there has been an increasing interest in the advances of photosynthetic biomaterials and therapies as described by numerous recently published reviews, where the potential of this novel concept is highlighted (19–22). However, and as stated by some of the aforementioned reviews, no previous study has demonstrated the safety of this approach in human patients. This gap represents a major obstacle for the translation of photosynthetic therapies into clinical practice.

In order to address this gap, in this work the safety and feasibility of using photosynthetic biomaterials was studied by implanting commercially available collagen scaffolds for dermal regeneration containing high concentrations of microalgae into human full-thickness skin wounds. Afterwards, tissue regeneration and several other relevant clinical and laboratory aspects were analyzed for up to 90 days, demonstrating for the first time that photosynthetic cells can be safely implanted in human patients, allowing tissue regeneration.

## Materials and Methods

### Photosynthetic Scaffold Fabrication

A cell-wall deficient *C. reinhardtii* strain (cw15-30-derived UVM4) was grown photomixotrophically at 20°C in sterile liquid Tris-Acetate-Phosphate medium (TAP) with constant agitation, and kept always in the exponential growth phase and constant white illumination (2500 lux, eq. 72.5 μE m^−2^ s^−1^) (23, 24). This particular strain of *C. reinhardtii* was chosen because our preliminary data showed its safety in *in vitro* and *in vivo* settings (13–16). Afterwards, photosynthetic scaffolds were fabricated following our previously optimized protocols, with minor modifications (14). Briefly, 25 cm^2^ scaffolds (Integra matrix, Integra Life Science Corporation) were slightly dried on a sterile gauze, and placed silicone-face down on a sterile cell culture plate. Then, 1.25× 10^8^ cells were resuspended in 850 μL of sterile TAP, and mixed in a 1:1 ratio with human fibrinogen (EVICEL^®^, Johnson & Johnson). Next, 850 μL of human thrombin (EVICEL^®^, Johnson & Johnson) were homogeneously added to the scaffolds, followed by the addition of the microalgae-fibrinogen mixture. Scaffolds were left undisturbed for 1 h to ensure complete polymerization, and then covered with 20 mL of sterile TAP. For quality control, biopsy samples were taken and suspended in 1 mL of TAP (Supplementary Table 1). During the microbiology testing period, scaffolds were left undisturbed at room temperature (RT) with constant white illumination for 4–6 days. Once negative results were confirmed, photosynthetic scaffolds were sterilely packaged and transported to the operating room.

### Photosynthetic Scaffold Characterization

Macroscopic imaging was performed using a standard stereoscope (Leica S6D) coupled to a digital camera. For SEM imaging, scaffolds were fixed in 2% glutaraldehyde and dehydrated with graded ethanol and final 100% acetone. Samples were then air-dried, sputtered with gold and analyzed using 5 kV (Hitachi TM3000). Metabolic activity of the photosynthetic scaffold was measured by introducing a 1 cm^2^ biopsy in the chamber of a Clark-type oxygen electrode (Oxygraph^+^ System, Hansatech Instruments) containing 1 mL of TAP. Afterwards, samples were subjected to dark/light (455 nm) cycles of 10 min, and the dissolved oxygen concentrations were recorded for 1 h.

### Clinical Study Design and Participants

This study is a single-center, first-in-human, early phase 1 clinical trial to assess the safety of photosynthetic biomaterials for the treatment of full thickness skin wounds (ClinicalTrials.gov identifier: NCT03960164, https://clinicaltrials.gov/ct2/show/NCT03960164). The clinical trial protocol was previously approved by the Research Ethics Committee of the Hospital del Salvador and the Metropolitan Health Service (approval number CECSSMO080820018). All patients involved in the study were selected according to the established criteria (Supplementary Table 2), and signed the informed consent form before inclusion in the study. During the study, local and systemic response of the treated patients were evaluated by several means as described below in this section as well as in **Figure 2**.

### Surgical Procedure and Clinical Follow Up

All surgical procedures performed in this study are standard for scaffold-dependent dermal regeneration approaches, and were performed in the operating room at Hospital del Salvador (Santiago, Chile), under general or spinal anesthesia and strict aseptic technique. To ensure wound sterility, microbiology testing of the wound bed was performed before photosynthetic scaffold implantation. For the patient scheduled for scare resection, no previous microbiology testing was performed. First, surgical debridement of the wound or scar removal, was performed, and the photosynthetic scaffolds were fixed to the defect with non-absorbable monofilament nylon 4/0 sutures (Ethilon^®^, Johnson & Johnson). For the first five patients, scaffolds were only covered with a transparent dressing (Tegaderm^®^, 3M) and sterile gauze. For the following patients the dressing system was optimized, thus scaffolds were covered with a flexible and transparent PDMS draining system and a NPWT dressing was placed on top (Renasys^®^, Smith & Nephew), leaving a window in the center to allow scaffold illumination (**Figure 4** and Supplementary Table 3). Finally, the illumination device (Supplementary Figure 1) was positioned on top of the dressing, and secured with elastic bandage. The control unit of the illumination device was handled by Bluetooth^TM^ and, to avoid over heating of the wound, the implanted scaffolds were subjected to dark/ light cycles of 10 and 20 min, respectively, for 7 days. Throughout the first 10 days, patients answered a self-evaluation questionnaire to evaluate pain intensity, burning, itching, smell, and light annoyance, using either visual analog scale (VAS) or Likert proportional scale.

Once adequate adherence of the scaffold to the wound bed was achieved (day 21), a second surgical procedure was performed in most patients (Supplementary Table 3). Here, an autologous partial skin graft was obtained with a dermatome (Acculan^®^ 3Ti, Aesculap) from the patient's thighs, and small serial fenestrations were created to avoid exudate accumulation. Then, the silicone sheet that covered the scaffold was removed and the autograft was sutured on top of the previously implanted photosynthetic scaffold, using non-absorbable monofilament nylon 4/0 sutures (Ethilon^®^). Finally, the autograft was secured with NPWT, and patients were hospitalized for 6 days. Prior to hospital discharge, the NPWT system was substituted for traditional advanced dressings and patients were kept under close outpatient follow-up for the next 90 days.

### Immune Cell Populations Analysis

Lymphocyte subpopulations from peripheral blood samples were measured by flow cytometry. Cells were stained with the following monoclonal antibodies against surface markers: CD3/CD16+CD56, CD19, CD8, and CD4 (BD Biosciences). Briefly, 5 μL of each antibody were added to 100 μL of blood samples, vortexed and incubated for 20 min at RT and in darkness. Next, 1 mL of BD FACS™ Lysing Solution (BD Biosciences) was added, and samples were centrifuged at 600 g, 4°C for 5 min. Supernatant was discarded, leaving 50 μL, and 1 ml of PBS+2% fetal bovine serum (ThermoFisher Scientific) was added to the samples and centrifuged at 600 g, 4°C for 5 min. Finally, supernatant was discarded leaving 50 μL, and 300 μL of 0.5% paraformaldehyde were added. Lymphocyte subpopulations were quantified by flow cytometry (FACSCanto II cytometer, BD Biosciences) using FACSDivaTM clinical software for data analysis.

### Cytokines Quantification

Serum from each patient was obtained by centrifugation of freshly isolated peripheral blood samples and stored at −80°C until analysis. The concentration of inflammatory cytokines in serum was determined by a CBA human inflammation kit (BD Biosciences) according to the protocol indicated by the manufacturer. This assay quantitatively measures TNF-α, IL-1β, IL-6, IL-8, IL-12p70, and IL-10 levels in a single sample. Briefly, capture beads and phycoerythrin-conjugated detection antibodies are incubated with the samples to form fluorescent sandwich complexes, which are measured by flow cytometry and compared to a calibration curve obtained with recombinant cytokines. The minimum detectable amount for the measured cytokines were as follows: TNF-α: 3.7 pg mL^−1^; IL-1β: 7.2 pg mL^−1^; IL-6: 2.5 pg mL^−1^; IL-8: 3.6 pg mL^−1^; IL-12p70: 1.9 pg mL^−1^; and IL-10: 3.3 pg mL^−1^.

### Clinical Laboratory Test

Hematological profiles, coagulation tests and biochemical profiles from whole blood samples were performed in all patients at the specific time points indicated in **Figure 2**. Hematological profile included hematocrit as well as erythrocytes, hemoglobin, platelets and leukocytes counts by certified clinical laboratory methods. Coagulation tests included international normalized ratio (INR), prothrombin time (PT) and partial thromboplastin time (PTT). Additionally, biochemical profiles included quantification of blood glucose, creatinine, bilirubin direct and total levels, serum glutamic-oxaloacetic transaminase (SGOT), serum glutamic-pyruvic transaminase (SGPT), alkaline phosphatase and C-reactive protein, as well as clinically relevant enzymatic activities and plasmatic electrolytes (sodium, potassium, and chloride). Profiles were performed by the clinical laboratory of the Hospital del Salvador according to their own standardize clinical protocols.

### Histopathology and Immunohistochemistry

Biopsy samples were obtained on days 7, 21, and 27 after photosynthetic scaffold implantation, fixed in a paraformaldehyde solution (4%), dehydrated in ethanol, and embedded in Paraplast (Leica Biosystems) at 60°C. Sections of 5 μm in thickness were cut and adhered to glass slides using 0.1% poly-L-Lysine (Sigma) and further dried at RT. Prior to the immunoreaction, some samples were stained with Hematoxylin-Eosin and Giemsa stain for morphological studies.

Immunohistochemistry was performed according to a previously established protocol (25). Briefly, sections were deparaffinized, rehydrated, and incubated with mouse monoclonal primary anti-CD68 (ThermoFisher Scientific) diluted 1:50, or rabbit polyclonal anti-CD31 (ThermoFisher Scientific) diluted 1:50, both in PBS containing 0.3% (v/v) Tween 20, overnight at 4°C. Nonspecific staining was blocked by 30 min immersion in Cas-Block solution (ThermoFisher Scientific) and goat serum (Gibco). After extensive rinsing in PBS, all sections were incubated for 1 h at RT with HRP-conjugated goat anti-mouse IgG (Rockland Immunochemicals) diluted 1:500 or HRP-conjugated goat anti-rabbit IgG (Sigma Aldrich) diluted 1:500 in PBS, respectively. The peroxidase reaction was visualized using the NovaRED kit (Vector Laboratories). After immunostaining, sections were slightly stained with Harris hematoxylin (Merck). For each immunohistochemical reaction, controls were performed by incubating the sections with PBS or by omitting the primary antibody. Sections were examined by standard light microscopy (Leica DM500) coupled to a digital camera.

### Statistical Analysis

Results were compared using the Kruskal–Wallis test and Dunn's post-test with GraphPad Prism 5 software. The Mann–Whitney *U*-test was employed for paired groups. *P* < 0.05 were considered statistically significant.

## Results

### Photosynthetic Scaffold Fabrication and Characterization

**Figure 1:**
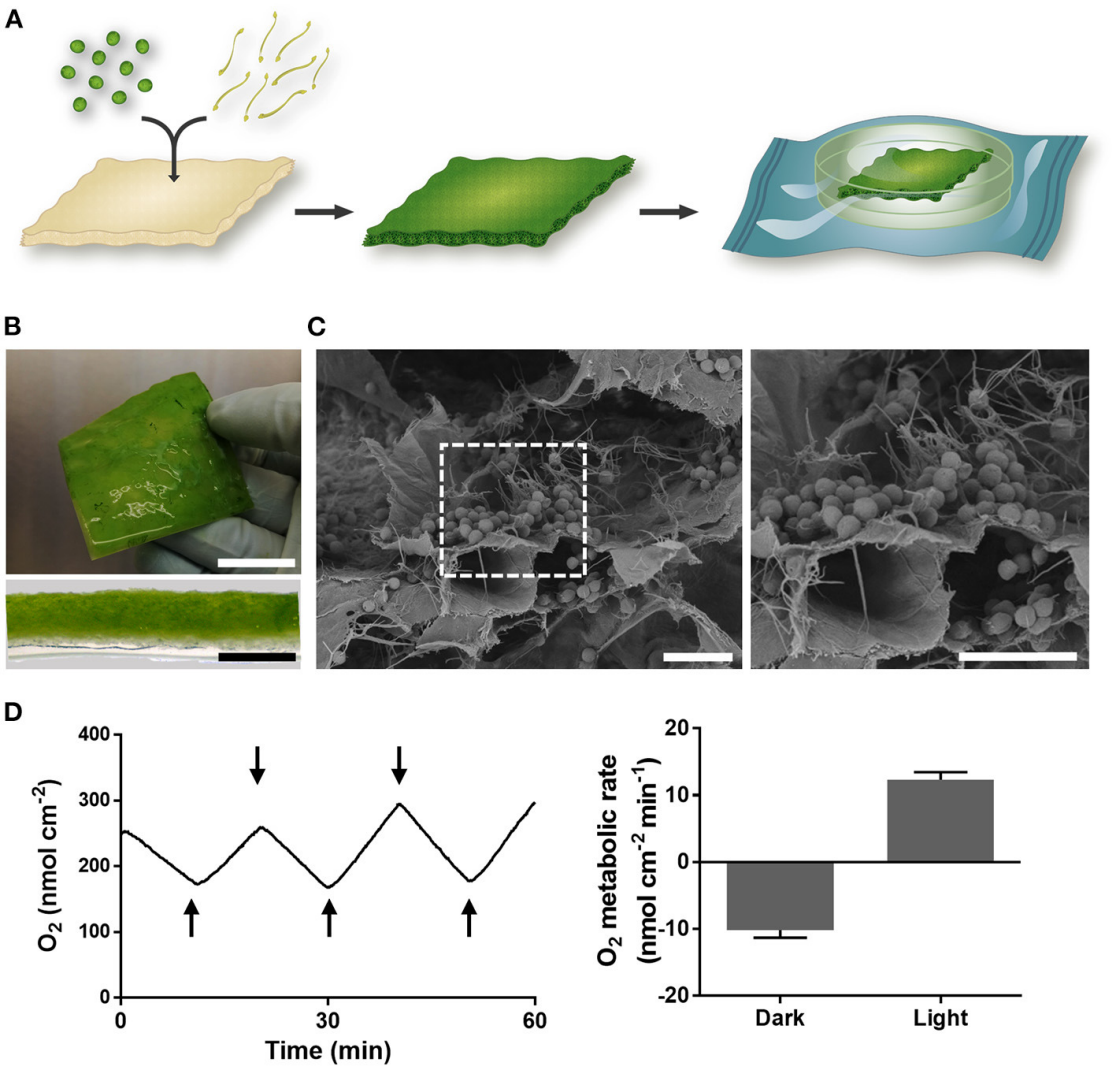
Photosynthetic scaffold fabrication and characterization. **(A)** Microalgae and fibrin were seeded in a commercially available collagen scaffold and allowed to grow during 4 days. Once sterility of the material was confirmed, scaffolds were packaged and delivered to the operating room. **(B)** Macroscopic image (top) and cross section (bottom) of photosynthetic scaffold. **(C)** SEM imaging showing microalgae embedded in the fibrin matrix inside the scaffold. **(D)** Representative evolution of oxygen concentration upon dark/light cycles of 10 min, represented by upper and lower arrows respectively (left), and scaffold metabolic rates (right). Scale bars represent 2 cm (**B**, top), 2 mm (**B**, bottom) and 20 μm **(C)**. Data in **(D)** is representative of at least five independent experiments and is expressed as mean value ± SEM (right).

For the fabrication of the photosynthetic scaffold, microalgae *C. reinhardtii* were cultured under sterile conditions, mixed with fibrin and incorporated in a commercially available scaffold for dermal regeneration (Figure 1A). Quality control of the scaffolds was performed (Supplementary Table 1), and were packed and delivered to the hospital for implantation.

Four days after microalgae seeding, imaging and metabolic characterization of the scaffold was performed. Macroscopic imaging showed homogeneous distribution of microalgae (Figure 1B), which was also confirmed by scanning electron microscopy (SEM), where microalgae were observed throughout the collagen scaffold embedded in a fibrin net (Figure 1C). Additionally, oxygen production of the scaffold was immediately detected upon light stimulation with an oxygen metabolic rate of 12.3 ± 1.1 nmol cm^−2^ min^−1^, while oxygen was consumed in the absence of light at a rate of 10.2 ± 1.1 nmol cm^−2^ min^−1^ (Figure 1D).

### Study Design and Participants

**Figure 2:**
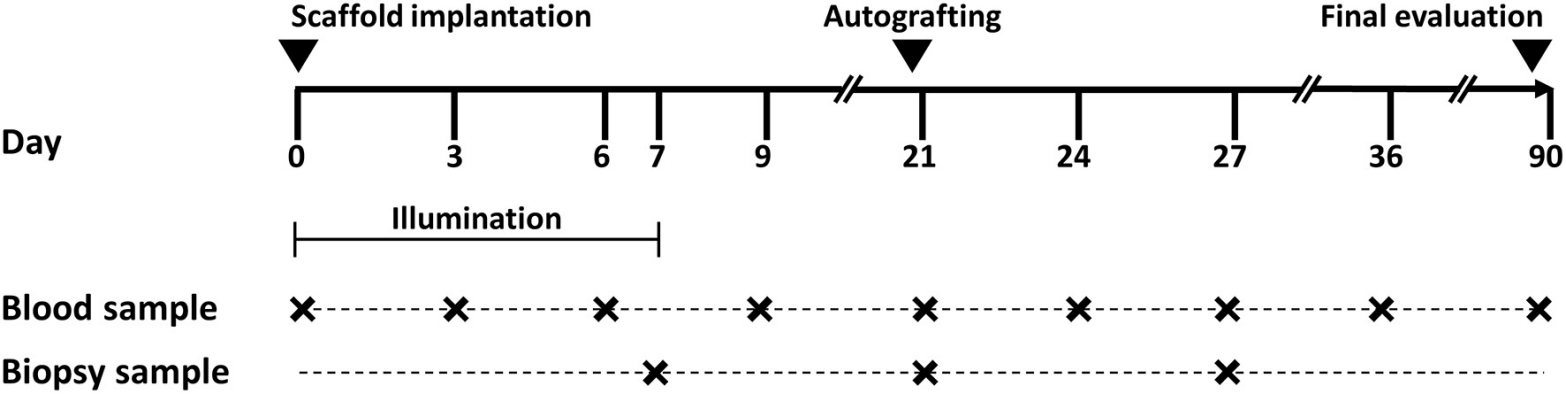
Study design. Photosynthetic scaffolds were implanted in patients previously recruited by Hospital del Salvador, Chile. Implanted scaffolds were illuminated during the first 7 days. On day 21 after implantation, an autologous split-thickness skin graft was surgically fixed over the previously implanted photosynthetic scaffold. All patients were evaluated and observed for up to 90 days. Blood and biopsy samples were taken at time points indicated in the figure.

Patients were selected according to specific inclusion/exclusion criteria (Supplementary Table 2). Once the wound bed was clean, photosynthetic scaffolds were implanted and illuminated for the following 7 days with a device specially designed for this study (Supplementary Figure 1). As these scaffolds are designed to provide a 3D matrix for dermal regeneration only, a second intervention was expected to be required to fully close the wound. Thus, on day 21, an autologous partial skin graft was performed on patients, except for some cases where this second intervention was not clinically required (Supplementary Table 3). Blood and biopsy samples were taken to assess the systemic and local immune response of the patients during the course of the study. Hematological and biochemical profiles, as well as the concentration of plasma cytokines and immune cells in peripheral blood were measured before scaffold implantation (day 0), before split skin autograft (days 3, 6, 9, and 21), and after split skin autograft (days 24, 27, 36, and 90). Additionally, for further histological and immunohistochemical analysis, biopsy samples were taken on days 7, 21, and 27 post-implantation. Due to sanitary restrictions caused by the COVID-19 pandemic or partial patient desertion, few tests were not performed for some patients at certain specific time points. A scheme of the study design is shown in Figure 2.

**Figure 3:**
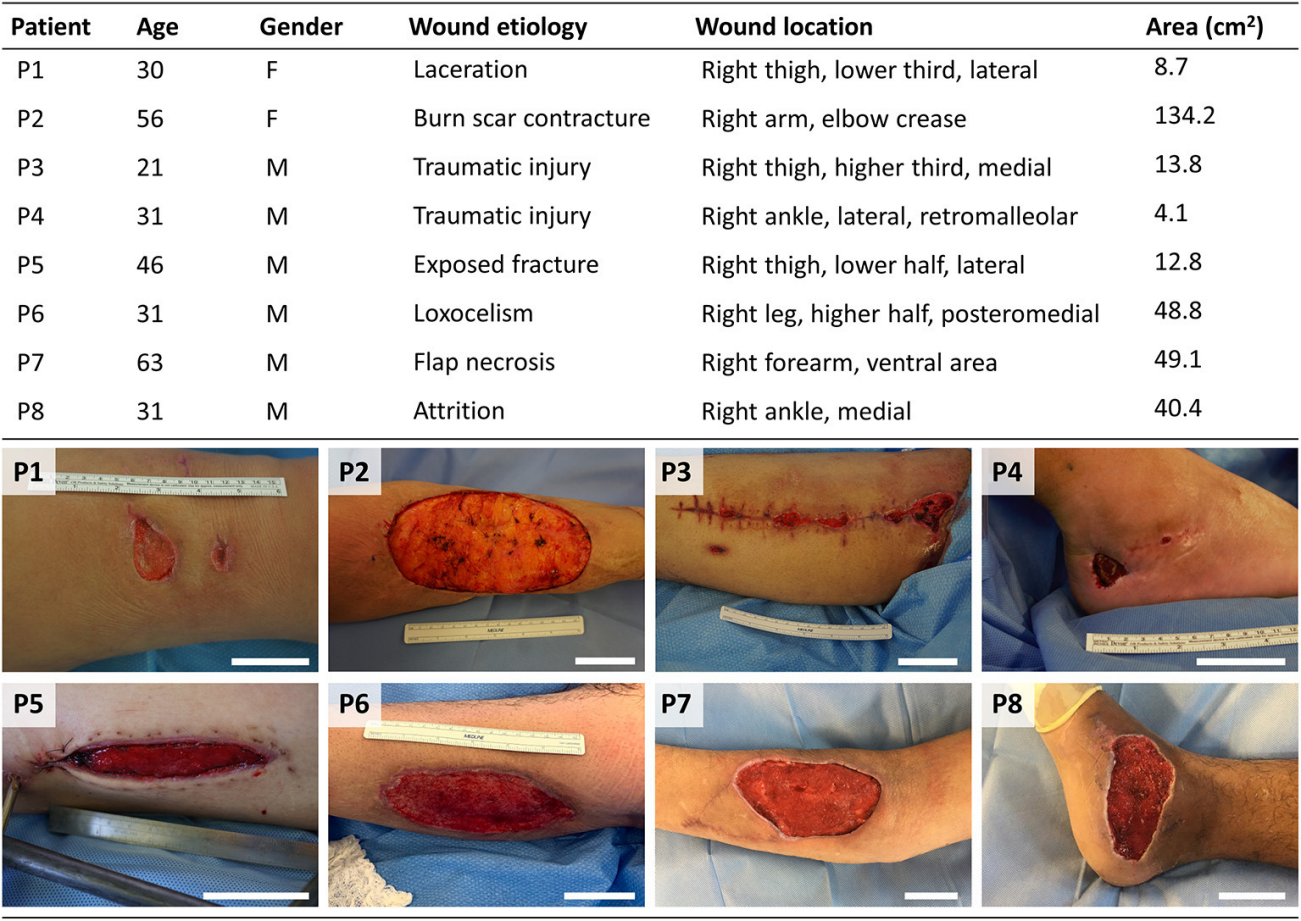
Characteristics of the enrolled patients. Table (top) showing characteristics of patients treated with the photosynthetic scaffold: patient's age (years), gender (Female/Male), etiology, location, and area of the wound. Images (bottom) show patient's full skin wounds at the moment of surgery, prior to photosynthetic scaffold implantation. Scale bars represent 5 cm.

A total of eight patients were enrolled in this study (Figure 3). Most wounds corresponded to single full-thickness skin defects, except for one patient (P3) who was treated for multiple wounds generated in the same event (Figure 3, bottom). The etiology of the wounds as well as the body location were diverse but, coincidentally, all wounds were located on the right extremities. The wound area was highly variable among patients, with a range of 4.1–134.2 cm^2^. Finally, the age of treated patients was broad, ranging from 21 to 63 years old.

### Photosynthetic Scaffold Implantation

**Figure 4:**
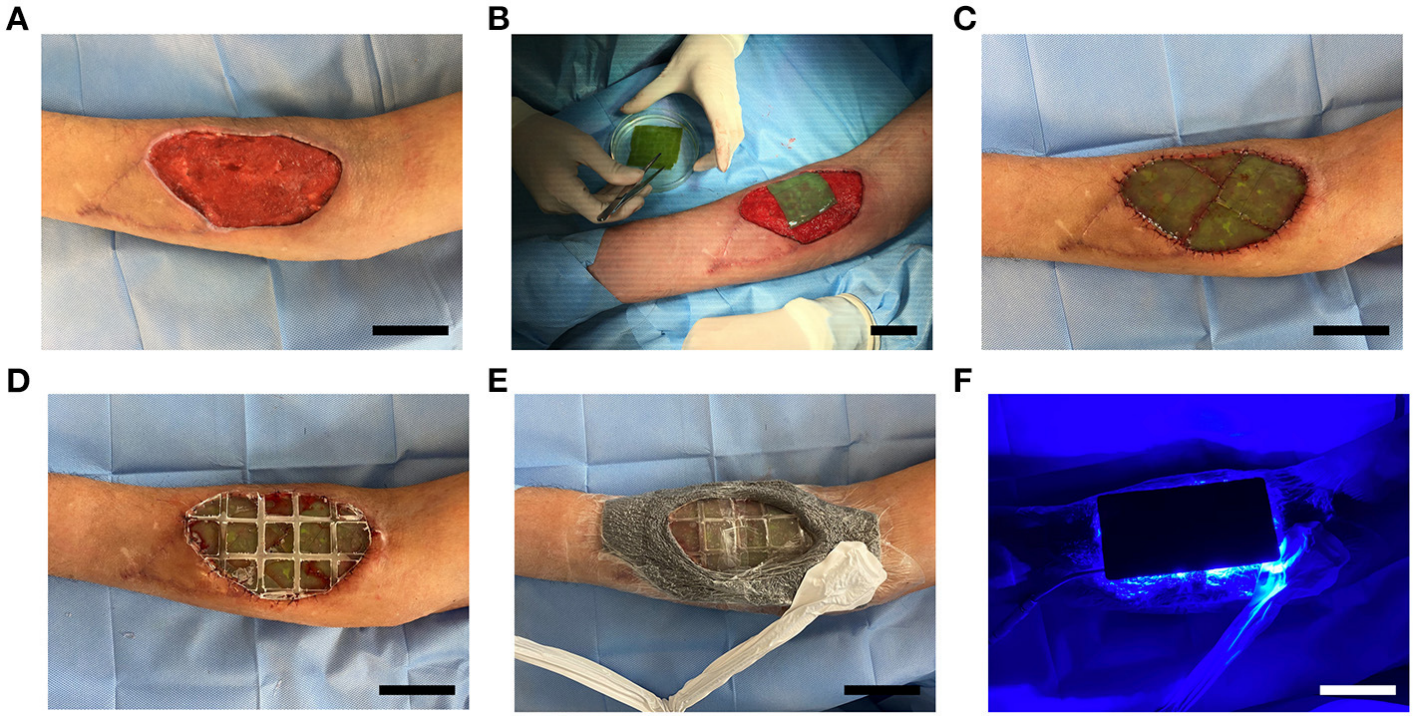
Photosynthetic scaffold implantation. Wound bed was cleaned and prepared for scaffold implantation **(A)**. Photosynthetic scaffolds were sutured between them and to the wound edges **(B,C)**, and covered with a flexible and transparent PDMS membrane **(D)**, which was then secured with negative pressure wound therapy, leaving a window over the scaffold to allow illumination **(E)**. Light device was then placed on top and illumination intensity was controlled **(F)**. Scale bars represent 5 cm.

All patients presented full-thickness wounds prior to scaffold implantation. Therefore, the overall surgical procedure was the same. However, some details had to be adapted among patients due to wound heterogeneity. As a representative example of the implantation process, the complete surgical procedure is shown for a single patient (P7; Figure 4). Prior to scaffold implantation, the wound bed was cleaned and prepared (Figure 4A). Photosynthetic scaffolds were then placed directly over the wound and trimmed as needed in order to perfectly fit the wound bed (Figure 4B). Surgical sutures were used to fix the scaffolds (Figure 4C). For patients P1–P5, scaffolds were covered with Tegaderm^®^ (3M) and sterile gauze, however, coverage was upgraded with the design of a flexible and transparent polydimethylsiloxane (PDMS) membrane with patterned channels to act as a draining system for patients P6–P8 (Supplementary Table 3), which was further secured with a negative pressure wound therapy (NPWT) dressing (Figures 4D,E). Finally, a light device specially designed for this study (Supplementary Figure 1) was placed on top to provide a specific and controlled illumination setting (Figure 4F).

For the patient shown in Figure 4, three 25 cm^2^ scaffolds were required to cover the wound area. Similarly, several scaffolds were also used for P2, P3, P6, and P8 in Table 1. A single 25 cm^2^ scaffold provided sufficient coverage of the entire wound in P1, P4, and P5.

**Table 1:** Hematological profile and coagulation test evaluated on patients with implanted photosynthetic scaffolds.

	**Sample day**									
**Parameter**	**0**	**3**	**6**	**9**	**21**	**24**	**27**	**36**	**90**	**Reference interval**
**Hematological profile**									
Hematocrit (%)	39.8 ± 5.8	39.2 ± 6.3	40.5 ± 5.7	37.4 ± 7.5	42.0 ± 5.6	39.7 ± 5.1	41.3 ± 4.8	40.2 ± 6.8	44.0 ± 7.1	**35.0–52.0**
Hemoglobin (g dL^−1^)	12.9 ± 2.4	12.6 ± 2.4	13.1 ± 2.2	12.1 ± 2.7	13.6 ± 2.2	13.0 ± 2.1	13.3 ± 2.0	13.1 ± 2.5	14.3 ± 2.9	**11.5–18.0**
Erythrocyte (10^6^ μL^−1^)	4.4 ± 0.6	4.4 ± 0.7	4.6 ± 0.6	4.2 ± 0.8	4.7 ± 0.5	4.5 ± 0.5	4.6 ± 0.5	4.5 ± 0.6	5.1 ± 0.5	**4.1–5.1**
Platelets (10^5^ μL^−1^)	3.4 ± 1.0	3.1 ± 0.8	3.2 ± 0.5	3.0 ± 0.6	3.0 ± 0.5	2.7 ± 0.4	2.7 ± 0.5	3.0 ± 0.4	2.8 ± 0.6	**1.4–4.0**
Leukocyte (10^3^ μL^−1^)	6.8 ± 1.2	6.6 ± 1.2	6.7 ± 1.2	7.4 ± 1.6	6.6 ± 1.0	7.0 ± 2.3	6.6 ± 1.4	6.9 ± 1.3	7.5 ± 2.1	**4.0–11.0**
Neutrophil (10^3^ μL^−1^)	3.7 ± 0.9	3.3 ± 0.8	3.4 ± 0.9	4.3 ± 1.4	3.3 ± 0.5	3.6 ± 1.5	3.5 ± 1.1	3.9 ± 1.0	4.0 ± 1.4	**2.5–6.3**
Lymphocyte (10^3^ μL^−1^)	2.1 ± 0.8	2.1 ± 0.7	2.1 ± 0.6	2.0 ± 0.8	2.4 ± 0.7	2.5 ± 0.8	2.2 ± 0.6	2.1 ± 0.7	2.7 ± 1.0	**0.8–3.6**
Monocyte (10^2^ μL^−1^)	6.4 ± 1.9	6.2 ± 2.1	6.5 ± 2.4	6.5 ± 2.1	6.0 ± 0.8	6.2 ± 1.4	6.2 ± 1.8	6.1 ± 1.9	6.4 ± 2.2	**2.0–6.0**
Eosinophil (10^3^ μL^−1^)	0.4 ± 0.3	0.5 ± 0.6	0.5 ± 0.5	0.5 ± 0.3	0.3 ± 0.2	0.3 ± 0.2	0.3 ± 0.2	0.3 ± 0.2	0.2 ± 0.1	**0.1–0.3**
Basophil (10^2^ μL^−1^)	0.0 ± 0.0	0.0 ± 0.0	0.0 ± 0.0	0.1 ± 0.0	0.0 ± 0.0	0.0 ± 0.0	0.0 ± 0.0	0.0 ± 0.0	0.0 ± 0.0	**0.0–2.0**
**Coagulation tests**										
INR	1.1 ± 0.1	1.1 ± 0.1	1.1 ± 0.1	1.1 ± 0.1	1.1 ± 0.0	1.1 ± 0.1	1.1 ± 0.1	1.1 ± 0.1	–	**0.9–1.3**
PT (s)	13.5 ± 1.1	13.2 ± 0.7	13.1 ± 0.5	13.2 ± 1.0	13.3 ± 0.5	13.2 ± 0.9	13.4 ± 0.9	13.2 ± 0.8	–	**12.0–18.0**
PTT (s)	30.7 ± 3.0	29.6 ± 2.9	31.4 ± 3.0	29.3 ± 3.1	28.2 ± 1.8	30.3 ± 3.5	31.6 ± 2.5	27.9 ± 1.5	–	**22.6–35.0**

*Values obtained before (day 0) and after photosynthetic scaffold implantation. Results are presented as mean value ± SD. N = 8 (day 0, 3 and 6); N = 7 (day 9); N = 6 (day 21, 24, 27, and 36); N = 4 (day 90). Reference intervals correspond to institutional reference ranges from Hospital del Salvador. Bold values correspond to the reference interval for normal (healthy) values*.

### Clinical Evolution and Patient Self-Evaluation

**Figure 5:**
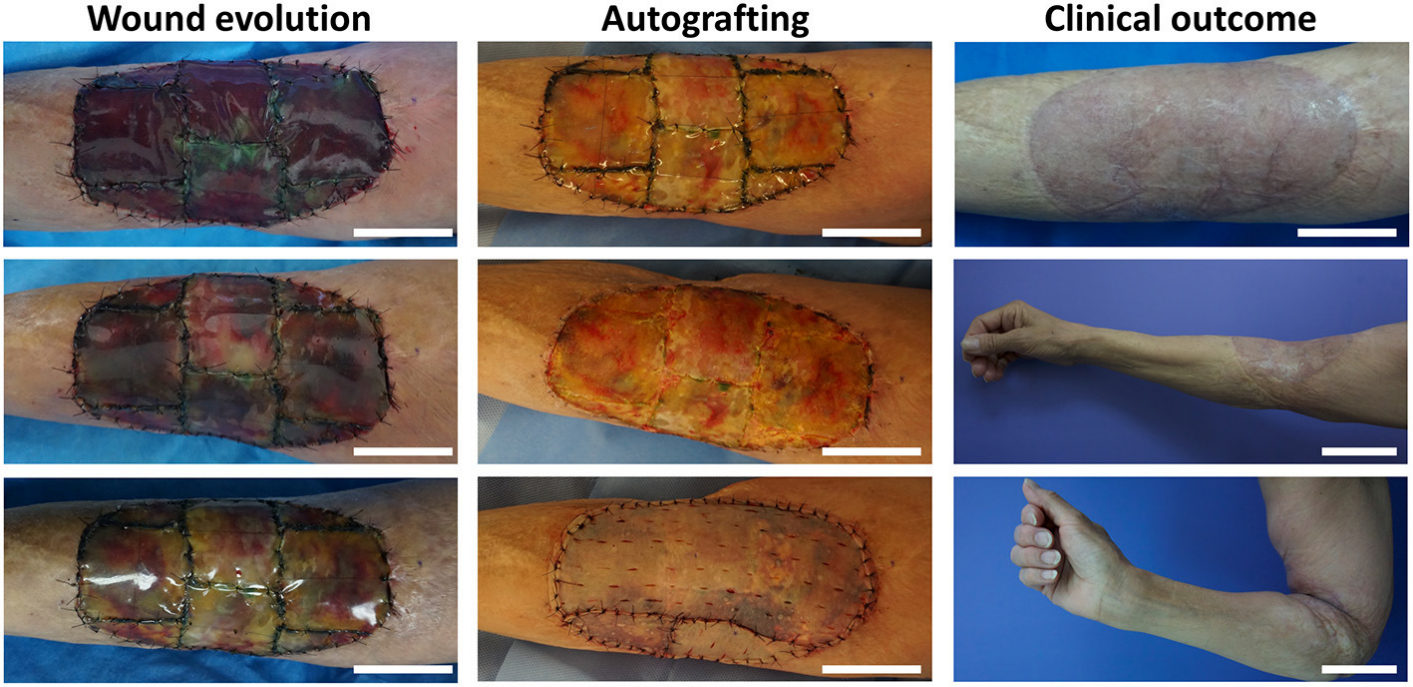
Overall wound evolution, autografting procedure, and clinical outcome. As a representative example, wound evolution of P2 is shown for days 1, 7, and 13 (from top to bottom). Autologous split-thickness skin graft was performed 21 days after scaffold implantation. Clinical outcome and functional recovery of the wound at day 90 post implantation are shown. Scale bars represent 5 cm except for Clinical outcome, middle and bottom, which represent 10 cm.

After scaffold implantation, clinical evolution was assessed over 90 days. As a representative example, the overall wound evolution of P2 is shown. Immediately after implantation, blood infiltration was observed in the photosynthetic scaffolds (Figure 5, Wound evolution, top). Proper adhesion and integration of the photosynthetic scaffold was also observed (Figure 5, Wound evolution). Notably, the contact area between the scaffold and the wound edge did not show clinical signs of local inflammation such as edema or erythema, at any time and in any patient in the surrounding healthy skin. At day 21 post surgery (Figure 5, Autografting), the silicone layer of the collagen scaffold was removed and autologous split-thickness skin graft was fixed to cover the wound in six out of the eight patients (Supplementary Table 3). Clinical outcome 90 days post scaffold implantation showed complete integration of the skin graft, no signs of morbidity and functional recovery of the wound area (Figure 5, Clinical outcome). The overall outcome of all patients was similar to P2, as all wounds closed at the expected time and, when corresponded, the autologous graft was integrated with the previously implanted scaffold. Nevertheless, some differences were observed among patients, specially in regard to the need of a second intervention. Moreover, it is worth to mention that a partial loss of the scaffold was seen in P4 due to patient misconduct.

**Figure 6:**
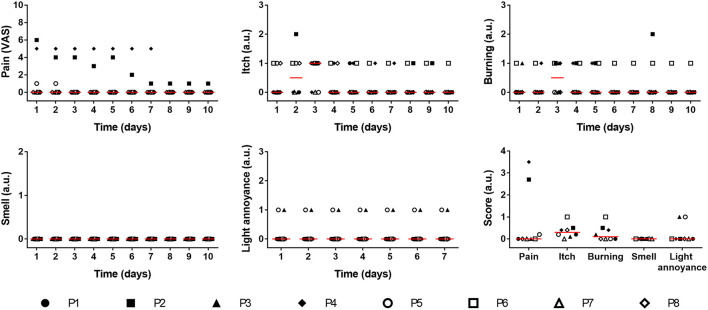
Self-evaluation of treated patients. After implantation of the photosynthetic scaffold several relevant parameters were evaluated by patients (P1–P8) for 10 days: pain intensity (expressed by the 0–10 visual analog scale), itch, burning (Proportional Likert scales 0–3) and smell (Proportional Likert scales 0–2). Light annoyance (Proportional Likert scales 0–3) was evaluated for 7 days. Average score for each parameter is also shown. Data is shown for each patient and red line represents the median. Kruskal–Wallis test was used, and no significant differences were observed between days.

Self-evaluation of the patients also supported the safety and feasibility of this photosynthetic approach (Figure 6). Here, five clinically relevant parameters were evaluated for up to 10 days after photosynthetic scaffold implantation. Visual analog scale (VAS) for subjective pain intensity (proportional 0–10 range) showed no pain (VAS ≤ 1) reported in six patients (P1, P3, P5, P6, P7, and P8) during the whole evaluation period, while one patient (P2) reported mid to high pain score (VAS = 6) at the first postsurgical day, which progressively decreased toward day 7. Another patient (P4) reported persistently mid pain scores (VAS = 5) during the 7 postsurgical days, and was unable to identify if the pain source was the subjacent calcaneus bone fracture or the implanted skin wound. Almost all patients reported low or no itching sensation in the implanted wounds. Burning sensation at the implantation site was reported to be absent or low (scores 0 or 1) during the evaluation time. Nevertheless, patient P2 reported a transient mild burning sensation at day 8 post-surgery. None of the patients reported any particular smell from the photosynthetic scaffold during the evaluation period, and only two of the patients (P3 and P5) reported a low light annoyance generated by the illumination device during all evaluation days. A summary of the self-evaluated scores for each parameter is shown in Figure 6 (bottom, right).

### Systemic Response After Photosynthetic Scaffold Implantation

**Figure 7:**
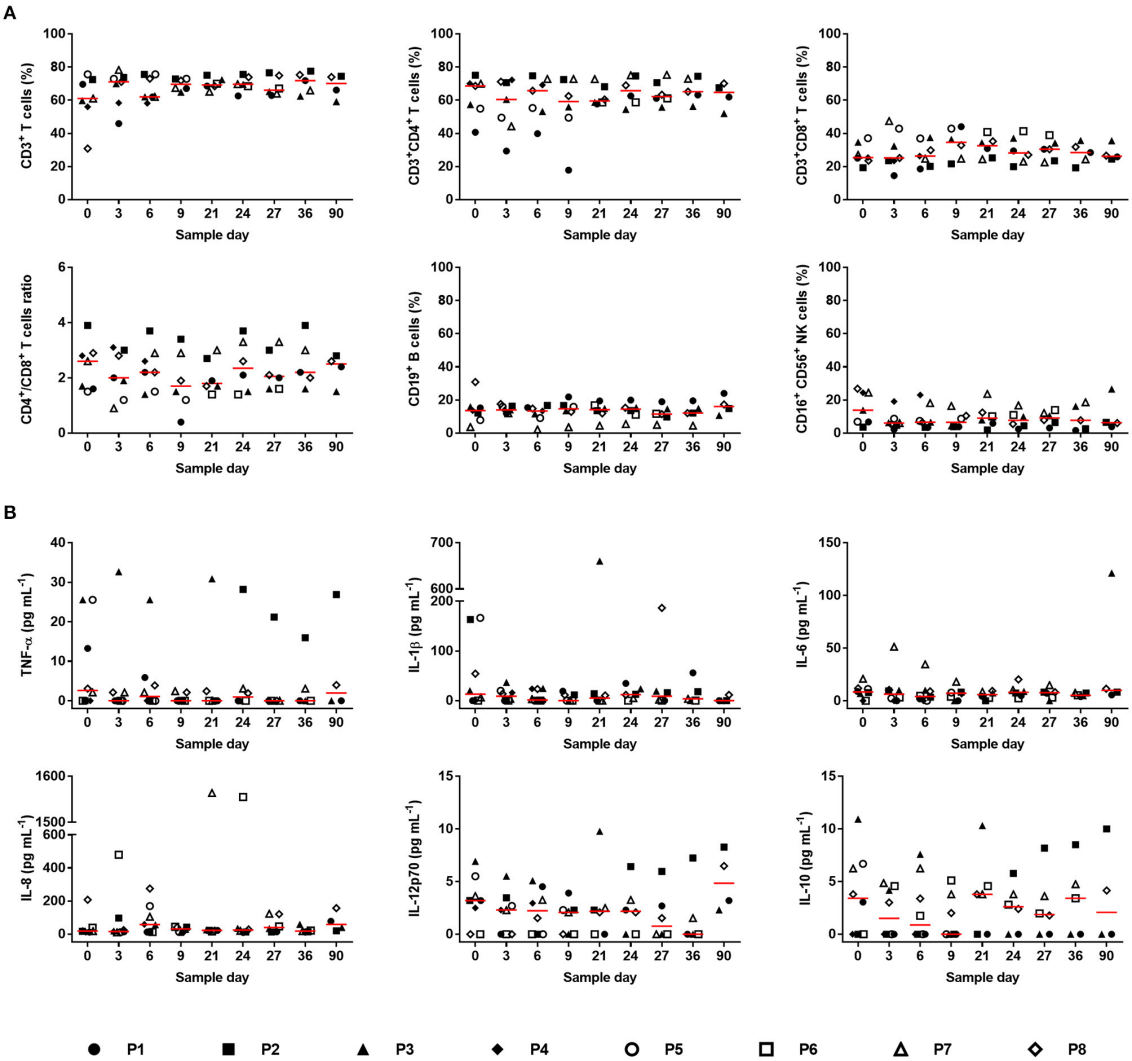
Systemic inflammatory response. **(A)** Lymphocyte subpopulations were evaluated before (day 0) and after scaffold implantation: T-cells (CD3^+^), T-helper cells (CD3^+^CD4^+^), Cytotoxic T-cells (CD3^+^CD8^+^), ratio (CD4^+^/CD8^+^), B-cells (CD19^+^) and NK cells (CD16^+^CD56^+^). Values for each patient and median (red line) are presented. **(B)** The concentration of inflammatory cytokines was determined before (day 0) and after photosynthetic scaffold implantation: tumor necrosis factor alpha (TNF-α), Interleukin-1 beta (IL-1β), Interleukin-6 (IL-6), Interleukin-8 (IL-8), Interleukin-12 (IL-12p70), and Interleukin-10 (IL-10). Values for each patient and median (red line) are presented. In **(A)**: *N* = 7 (day 0, 3, and 6), *N* = 6 (day 9, 21, 24, and 27), *N* = 5 (day 36), *N* = 4 (day 90). In **(B)** *N* = 8 (day 0, 3, and 6), *N* = 7 (day 9), *N* = 6 (day 21, 24, 27, and 36), *N* = 4 (day 90). Kruskal–Wallis test was used, and no significant differences were observed between days.

The systemic inflammatory response to the photosynthetic scaffold was analyzed in detail in this work. Lymphocyte subpopulations and levels of inflammatory cytokines present in plasma were evaluated. Total percentage of T-cells (CD3^+^), T-helper cells (CD3^+^CD4^+^) and cytotoxic T-cells (CD3^+^CD8^+^) or their ratio (CD4^+^/CD8^+^) did not increase after surgical procedure (Figure 7A), showing that the photosynthetic implant did not trigger neither a Th1 nor Th2 immune response. Similar results were obtained for B-cells (CD19^+^) and NK cells (CD16^+^CD56^+^), confirming that the Th2 immune response was absent. Comparable results were observed for six circulating inflammatory cytokines (TNF-α, IL-1β, IL-6, IL-8, IL-10, and IL-12p70) where no significant changes could be detected as a consequence of the photosynthetic treatment (Figure 7B).

Additionally, blood samples were taken according to the time points shown in Figure 2, and critical hematological (Table 1) and biochemical (Table 2) parameters were assessed for up to 90 days. Hematological results are summarized in Table 1. No significant differences were observed in the mean values of hematocrit percentage, hemoglobin concentration and erythrocytes count. Similarly, the number of platelets did not vary along with the treatment. No significant changes were detected in the total number of leukocytes or in specific subpopulations of neutrophils, lymphocytes, and basophils. All the parameters mentioned above were found normal within the institutional reference values during the 90 days follow-up. However, eosinophil counts were above the normal range from days 0 to 9 before decreasing within normal limits. Besides, coagulation tests were performed, which did not show significant changes compared to pre-implantation of the photosynthetic scaffold (day 0). Plasmatic electrolytes, i.e., sodium, potassium, and chloride, were evaluated before (day 0) and up to 90 days post-implantation of the photosynthetic scaffold, and no significant changes or values outside the institutional reference range were detected during the evaluation period. Once again, no changes nor values outside institutional reference ranges were detected within the biochemical profile, except for C-reactive protein which exceeded normal limits on day 0 (pre-scaffold implantation) and days 3 and 6 post implantation, and decreased on the following time points returning within normal institutional reference ranges (Table 2).

**Table 2:** Plasmatic electrolytes and biochemical profile.

	**Sample day**									
**Parameter**	**0**	**3**	**6**	**9**	**21**	**24**	**27**	**36**	**90**	**Reference interval**
**Plasmatic electrolytes**										
Sodium (mmol L^−1^)	140.1 ± 1.7	139.5 ± 1.8	139.9 ± 1	141.6 ± 2.7	140.2 ± 2.9	138.8 ± 4.4	140.0 ± 2.6	140.0 ± 2.8	142.0 ± 3.4	**136.0–145.0**
Potassium (mmol L^−1^)	4.6 ± 0.2	4.5 ± 0.3	4.4 ± 0.3	4.4 ± 0.4	4.3 ± 0.3	4.1 ± 0.3	4.4 ± 0.2	4.6 ± 0.5	4.5 ± 0.5	**3.5–5.1**
Chloride (mmol L^−1^)	104.3 ± 3.0	103.1 ± 2.4	102.6 ± 1.5	105.3 ± 4.0	103.0 ± 3.9	102.7 ± 4.0	102.7 ± 2.8	103.5 ± 3.7	103.8 ± 1.3	**98.0–107.0**
**Biochemical profile**										
Glucose (mg dL^−1^)	89.4 ± 6.9	87.7 ± 19.6	88.5 ± 19.1	94.0 ± 18.1	83.0 ± 10.6	78.0 ± 12.1	84.2 ± 11.1	85.8 ± 7.5	102.4 ± 30.7	**70.0–105.0**
Creatinine (mg dL^−1^)	0.9 ± 0.1	0.9 ± 0.2	0.9 ± 0.1	0.9 ± 0.2	0.9 ± 0.2	0.9 ± 0.2	0.9 ± 0.2	0.8 ± 0.1	0.9 ± 0.1	**0.6–1.1**
Bilirubin direct (mg dL^−1^)	0.2 ± 0.0	0.2 ± 0.0	0.2 ± 0.1	0.2 ± 0.1	0.2 ± 0.1	0.2 ± 0.1	0.2 ± 0.1	0.2 ± 0.1	0.3 ± 0.2	**0.0–5.0**
Bilirubin total (mg dL^−1^)	0.5 ± 0.1	0.4 ± 0.1	0.4 ± 0.1	0.4 ± 0.2	0.6 ± 0.1	0.5 ± 0.2	0.4 ± 0.2	0.4 ± 0.1	0.7 ± 0.5	**0.2–1.2**
SGOT (IU L^−1^)	23 ± 8.8	18.8 ± 5.1	27.3 ± 10.5	42.2 ± 22.6	22.2 ± 3.9	15.8 ± 4.2	19.5 ± 3.8	22.0 ± 11.9	26.3 ± 18.7	**5.0–34.0**
SGPT (IU L^−1^)	38.6 ± 19.7	31.8 ± 11.2	46.6 ± 21.7	70.2 ± 34.9	34.8 ± 10.2	23.2 ± 9.2	28.0 ± 10.0	32.6 ± 33.0	42.3 ± 44.6	**0.0–55.0**
Alkaline phosphatase (IU L^−1^)	109.3 ± 71.2	101.6 ± 61.2	104.9 ± 54.2	85.7 ± 18.5	85 ± 12.4	73.8 ± 12.8	79.8 ± 18.4	85.5 ± 14.7	93.5 ± 19.6	**40.0–150.0**
C-reactive protein (mg L^−1^)	6.1 ± 6.5	6.3 ± 4.4	6.5 ± 7.8	4.4 ± 4.4	1.8 ± 1.5	2.4 ± 2.0	3.1 ± 2.5	4.0 ± 3.5	4.7 ± 5.6	**0.0–5.0**

*Values obtained before (day 0) and after photosynthetic scaffold implantation. Results are presented as mean value ± SD. N = 8 (day 0, 3, and 6); N = 7 (day 9); N = 6 (day 21, 24, 27, and 36); N = 4 (day 90). SGOT, serum glutamic-oxaloacetic transaminase; SGPT, serum glutamic-pyruvic transaminase. Reference intervals correspond to institutional reference ranges from Hospital del Salvador. Bold values correspond to the reference interval for normal (healthy) values*.

### Local Response After Photosynthetic Scaffold Implantation

**Figure 8:**
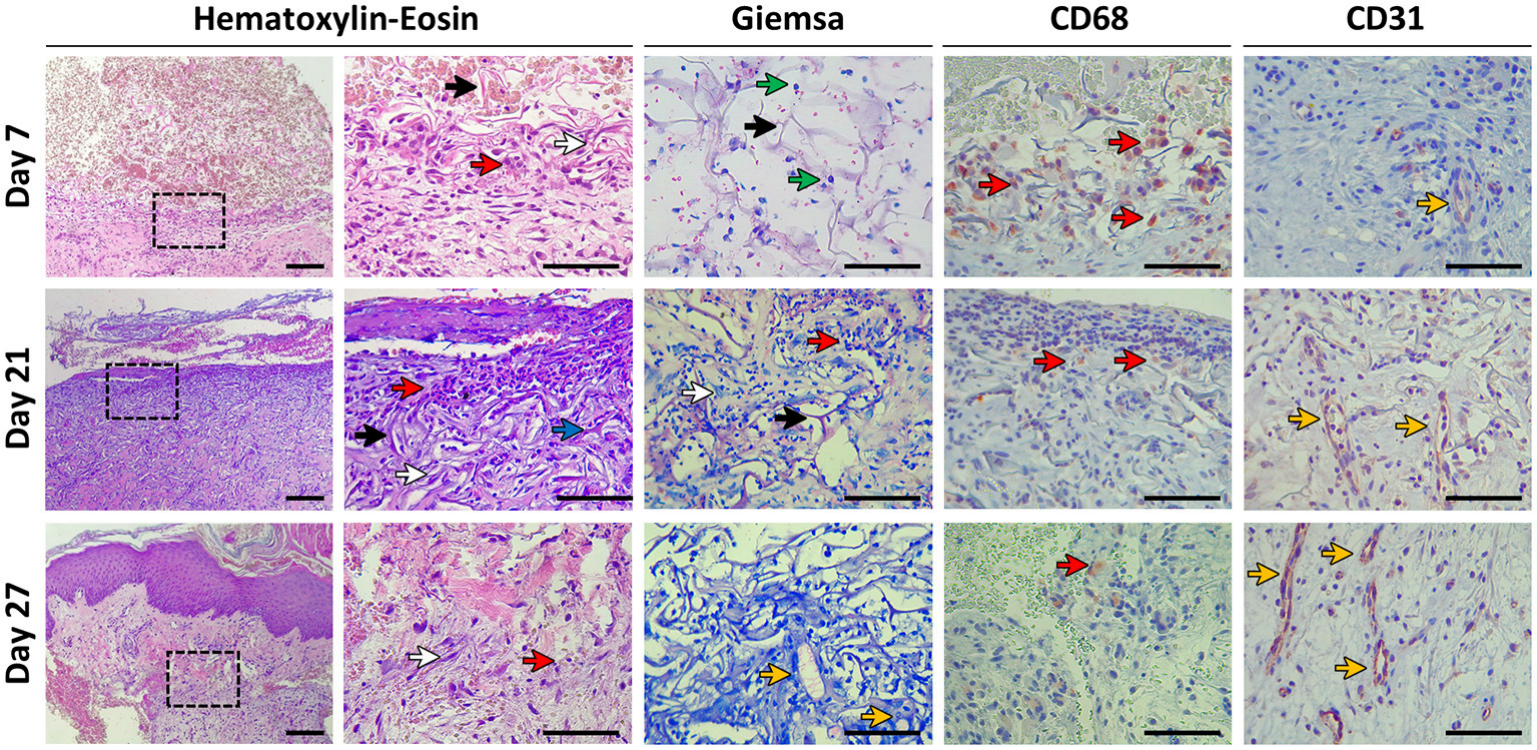
Histological evaluation. Biopsies were taken at days 7, 21, and 27 post-scaffold implantation. Histomorphology was evaluated with Hematoxylin-Eosin and Giemsa staining. Immunohistochemistry was performed to study the presence of macrophages (CD68) and endothelial cells (CD31). Colored arrows indicate: collagen fibers (black arrows), fibroblasts (white arrows), immune cells (red arrows), fibrin deposits (blue arrow), microalgae (green arrows), and vascular structures (yellow arrows). The indicated area in the left column of Hematoxylin-Eosin is magnified in the right column. Scale bars represent 100 μm for all pictures except for Hematoxylin-Eosin left column which represents 200 μm. All samples correspond to P8, except for Giemsa (P1).

In order to determine the local effect of the photosynthetic scaffolds, histological assays were performed at the times described in Figure 2. On day 7 after scaffold implantation, Hematoxylin-Eosin (HE) staining clearly identifies of the implanted photosynthetic scaffold (Figure 8, Hematoxylin-Eosin). Higher magnification of the biopsy reveals randomly oriented collagen fibrils, immune cells and fibroblastic cells with scarce cytoplasm and heterochromatic, flattened or spindle-shaped nucleus. Additionally, erythrocyte infiltration was also observed throughout the biopsy sample. HE staining on day 21 reveals an organized dermis. Higher magnification reveals randomly oriented collagen fibrils with some fibrin deposits, fibroblastic cells and immune cells. On day 27, which corresponds to six days after autologous split-thickness skin graft, dermo-epidermal structure can be clearly distinguished with the presence of the stratum corneum. Additionally, higher magnification shows complete integration between autologous tissue and patient's neodermis. At this time point, the presence of immune cells decreased, while fibroblast cells were immersed in a fibrillar collagen matrix.

In Giemsa staining (Figure 8, Giemsa), the presence of microalgae was observed on day 7 showing typical size, morphology and bluish granular cytoplasm. Infiltrated erythrocytes were also observed on day 7. Microalgae were not detected at days 21 and 27. Neovascularization oriented toward the photosynthetic scaffold could be observed, with the presence of multiple early blood vessels embedded in normal extracellular matrix and moderate presence of spindle-shaped fibroblasts, shaping a normal dermo-epidermic histoarchitecture.

Immunohistochemistry with macrophage marker CD68 (Figure 8, CD68) showed the presence of these immunocompetent cells, both in the photosynthetic implant and in the dermis adjacent to the implant. Macrophages were mainly distributed in the border region of the patient's dermis with the photosynthetic matrix. On day 7, a clear presence of these cells was observed, while on days 21 and 27, isolated macrophages were observed throughout the samples. Fused macrophages and foreign body giant cells were not detected at any time point.

By means of CD31 antibody immunohistochemistry (Figure 8, CD31), it was possible to appreciate the organization of capillaries and blood vessels oriented toward the region of the photosynthetic implant. On day 7, the presence of endothelial cells in the process of angiogenesis was observed, that is, mostly without a vascular lumen, however with a clear distribution in the dermal tissue immediately adjacent to the implant. The presence of endothelial cells and vascular structures in the area of the photosynthetic scaffold was evidenced in samples from days 21 and 27.

## Discussion

Oxygen plays a key role in most steps involved in wound healing and tissue regeneration (26). Among others it is required for energy production, as a signal transduction molecule and as antibiotic (27). Thus, poor oxygenation is one of the main contributing factors to non-healing in chronic wounds (1). Due to the lack of appropriate vascularization, oxygen delivery is a major drawback for current biomaterial-based technologies in regenerative medicine, representing an active field in biomedical research (5). Among novel approaches, the implantation of photosynthetic cells into tissues represent a promising alternative method to deliver oxygen in a local and controlled manner (19), but no clinical data is available to support this approach. Hence, in the present work, the safety of this novel concept was studied for the first time by implanting photosynthetic scaffolds into full skin wounds in human patients.

After implanting photosynthetic scaffolds, macroscopic analysis of all wounds did not show signs of inflammation, such as edema or erythema, at any time point, being consistent with the patient's self-evaluation, where no relevant levels of discomfort were reported. Similarly, no clinical signs of adverse effects were detected as evidenced by general laboratory analysis. Here, it is worth to mention that a control group without microalgae was not included in this study because the scaffold and fibrin are broadly described to be safe for clinical use.

One of the most valuable results of this trial was the low immune responses observed against the foreign implanted microalgae. Quantitative differences in the total and subpopulations counts of leukocytes is a well-known parameter to detect immune cellular responses during exposition to biomaterials, thus adverse reactions are characterized by an increased count of CD3^+^, CD4^+^, and T-cells (28). However, such changes were not detected in any of the treated patients, showing that the implantation of photosynthetic scaffolds did not affect the circulating lymphocyte subpopulations. Additionally, healthy adults with no inflammation or alteration in their immune system present a ratio of CD4^+^/CD8^+^ T cells equal to 2, and should be always higher than 1 (29), which was also observed for patients at all time points. Similar results were observed for inflammatory cytokines, where an increase of 3 to 4 orders in magnitude are described to be triggered by pathogen-related stimuli (30), but no significant difference in response to the microalgae implantation was detected.

The immune tolerance observed here to *C. reinhardtii*, agreed with our previous results reported in zebrafish (31) and mice (14, 15), and was consistently evidenced by different means in all subjects of this study at all the analyzed time points. Fibrin coating of the algae in the scaffold could only partially explain this result because its half-life *in vivo* is estimated to be ~10–14 days (32), hence a later reaction could have been expected in any case. In contrast to other microorganisms, *C. reinhardtii* have no pathogenic or toxicogenic potential, and have been granted with a GRAS (Generally Recognized as Safe) status by the US FDA (33). In fact, the critical pathogen associated molecular patterns that are recognized by the native immune system (e.g., LPS or single strain RNA) have not been described to be present in *C. reinhardtii*. Therefore, an interesting option to consider is that the human immune system could have not evolved in the need to recognize such kind of cells as foreign entities. This particular feature of immune tolerance should be explored in more detail, and could have an impact in other medical fields, where these photosynthetic cells could be used not only to provide oxygen into local tissue sites, but also to release other therapeutic molecules such as recombinant proteins (15, 17, 34).

Furthermore, a histological analysis showed coexisting microalgae and infiltrated cells in the collagen scaffold at day 7 but not at day 21, which was expected based on our previous reports (15). Overall, infiltration of fibroblasts, macrophages, and neovascularization was observed in the analyzed samples, suggesting excellent integration between the photosynthetic scaffold and the wound bed. These characteristics correspond to those expected in skin wound reparative processes, and are critical for the success of the split skin autograft, which was observed histologically on day 27, where a complete integration between the autologous split-thickness skin graft and the regenerated neodermis of the patient was observed. This is interesting when compared to allografts, which have a limited persistence *in vivo*, as immune rejection usually occurs within a few days or weeks in the case of patients with suppressed immune response or even less in the case of pig xenografts (35, 36), leading to destruction of the graft carried out predominantly by CD8^+^ T cells (37).

Aiming to discard potentially phototoxic effects in the regenerative process (38), the feasibility of illuminating wounds for extended periods under safe and controlled conditions was also confirmed. Here, the illumination setting was chosen according to our preliminary *in vivo* data, where microalgae were found to be alive at day 7-post implantation but not at day 14 (15). Nevertheless, survival of the implanted microalgae in humans is a critical issue to be studied in further research. In order to stimulate the photosystem II, a novel LED-based device was designed with an emission wavelength of 455 nm. Red light (623 nm) is also absorbed by chlorophyll; however, it was discarded in this study for its side effect of increasing local heat. Besides photons, a new generation of illumination devices could include integrated chemical and metabolic sensors to control light emission, and thereby control oxygen production. For instance, desired oxygenation levels in the regenerating tissue could be spatiotemporally achieved by coupling lighting intensity to local oxygen sensors in the photosynthetic biomaterials. In order to improve scaffold illumination, wound dressings were optimized during the study. Thus, the last three patient's wounds were covered with a flexible and transparent PDMS membrane which, in contrast to standard gauze, allowed full light penetration and scaffold illumination. Additionally, because such PDMS membranes were patterned with channels, together with NPWT, this dressing acted as an exudate draining system, avoiding exudate accumulation as well as opacity of the lighting interphase.

Although to evaluate the efficacy of photosynthetic biomaterials was out of the scope of the present work, it was clear that the presence of the microalgae allowed key regenerative processes such as cell migration, ECM deposition, and neovascularization. Thus, the results presented here show for the first time the safety of photosynthetic biomaterials for human treatment. These results could be extremely relevant for the translation of photosynthetic therapies into clinics, but further studies need to be done in order to confirm this in a larger population of patients and against appropriate controls, such as standard of care. Before testing efficacy, the safety of photosynthetic biomaterials should be studied in hypoxic chronic wounds, which are normally present in compromised patients. Additionally, since clinical outcomes may vary between individuals, pathologies, or tissues, studies for efficacy will have to consider the optimization of crucial aspects such as algae density, type of injury and illumination settings. Finally, the safety of genetically engineered microalgae should also be explored to further evaluate potential synergistic effects between the simultaneous release of oxygen and recombinant therapeutic agents that may contribute to tissue regeneration (39, 40). In conclusion, this study represents the first in human clinical trial to prove the safety of implanting microalgae as an approach to oxygenate tissues by photosynthetic therapy. The implantation of photosynthetic scaffolds in eight patients with full thickness skin wounds did not trigger any local or systemic immune response within the 90 days follow-up, and allowed full tissue regeneration. These results will significantly help to translate photosynthetic biomaterials and therapies into clinical settings, and will contribute to the understanding of potential symbiotic relationships between humans and photosynthetic cells. This novel concept of human photosynthesis is intriguing and could have enormous translational applications, with an impact far beyond tissue engineering and regeneration.

## Data Availability Statement

Data available upon request due to privacy/ethical restrictions.

## Ethics Statement

The studies involving human participants were reviewed and approved by Research Ethics Committee of the Hospital del Salvador and the Metropolitan Health Service (approval number CECSSMO080820018). The patients/participants provided their written informed consent to participate in this study. Written informed consent was obtained from the individual(s) for the publication of any potentially identifiable images or data included in this article.

## Author Contributions

JTE, WC, JPC, and MLO conceived the clinical trial. JTE, AE-Z, and WC supervised the clinical trial. XM and CW obtained biological samples and wound cures. MB, RC-O, and FC carried photosynthetic scaffold fabrication, oximetry, and total quality controls. CDG designed, built, and tested the illuminating device. VS and MRB carried out flow cytometry and data processing and interpretation. SSM and JV planned, performed and interpreted histology, and immunohistochemistry. JTE, MB, RC-O, FC, MLO, JPC, and AE-Z analyzed clinical and research data. JTE, MB, RC-O, FC, MLO, JPC, and AE-Z wrote the paper. All authors contributed, reviewed, and approved the final version of this manuscript.

## Funding

This work was supported by CORFO Portafolio I+D grant 18PIDE98887, FONDECYT 1200280, and FONDEQUIP/EQM 140016.

## Conflict of Interest

JTE is co-founder of SymbiOx Inc., a startup company that owns IP for the technology described here. During the conduct of the trial, MB, RC-O and FC were full-time employees of SymbiOx Inc., while AE-Z, XM and CW were part-time employees. All SymbiOx team members were financed with a R&D grant obtained from the Chilean Ministry of Economics (CORFO 18PIDE98887). The remaining authors declare that the research was conducted in the absence of any commercial or financial relationships that could be construed as a potential conflict of interest.

## Publisher's Note

All claims expressed in this article are solely those of the authors and do not necessarily represent those of their affiliated organizations, or those of the publisher, the editors and the reviewers. Any product that may be evaluated in this article, or claim that may be made by its manufacturer, is not guaranteed or endorsed by the publisher.
