# Supplementary material for: A First in Human Trial Implanting Microalgae Shows Safety of Photosynthetic Therapy for the Effective Treatment of Full Thickness Skin Wounds
Source: Front Med (Lausanne). 2021 Nov 30;8:772324. doi: 10.3389/fmed.2021.772324 (PMC8669306; doi:10.3389/fmed.2021.772324)
Supplement: Supplementary file 1 [file Data_Sheet_1.pdf]

Supplementary Material

1 Supplementary Data

1.1 Illumination device

A device was designed for the controlled illumination of implanted photosynthetic scaffolds, composed of a control unit and a lighting system (Andes Scientific Instruments, Sky-Walkers SpA, Litueche, Chile). Electronics of the lighting system are based on Pulse Width Modulation (PWM) intensity control of blue LEDs (wavelength 455 nm) through an electronic driver. LEDs were soldered on a printed circuit board (PCB) and connected through flat ribbon cables, all supported by a copper sheet with a living hinge pattern. The electronics and rechargeable lithium-ion batteries were held in a portable control unit with standard fixing to the institutional clinical holders. A user interface allowed programming of the LED intensity from 0 to 100% (from a maximal 550 mW per LED) and light scheduled via a Bluetooth™ application for remote control of the device. An LCD screen continuously displays the battery state and temperature of the electronic driver, the copper sheet and the batteries.

1.2 PDMS membrane fabrication

A polydimethylsiloxane elastomer (PDMS; SYLGARD™ 184 Silicone Elastomer Kit, Dow) membrane was fabricated to serve as a draining system for patients in the study. Patient's wounds were measured and areas were calculated (shown in Fig. 3). An 8 mm in thickness PDMS membrane was then fabricated for each patient, by curing PDMS elastomer following manufacturer's instructions. A special mold containing a grid pattern was used, providing draining channels to the PDMS membrane once cured. PDMS was then autoclaved to ensure sterilization, and delivered to the operating room together with the photosynthetic scaffolds.

2 Supplementary Figures and Tables

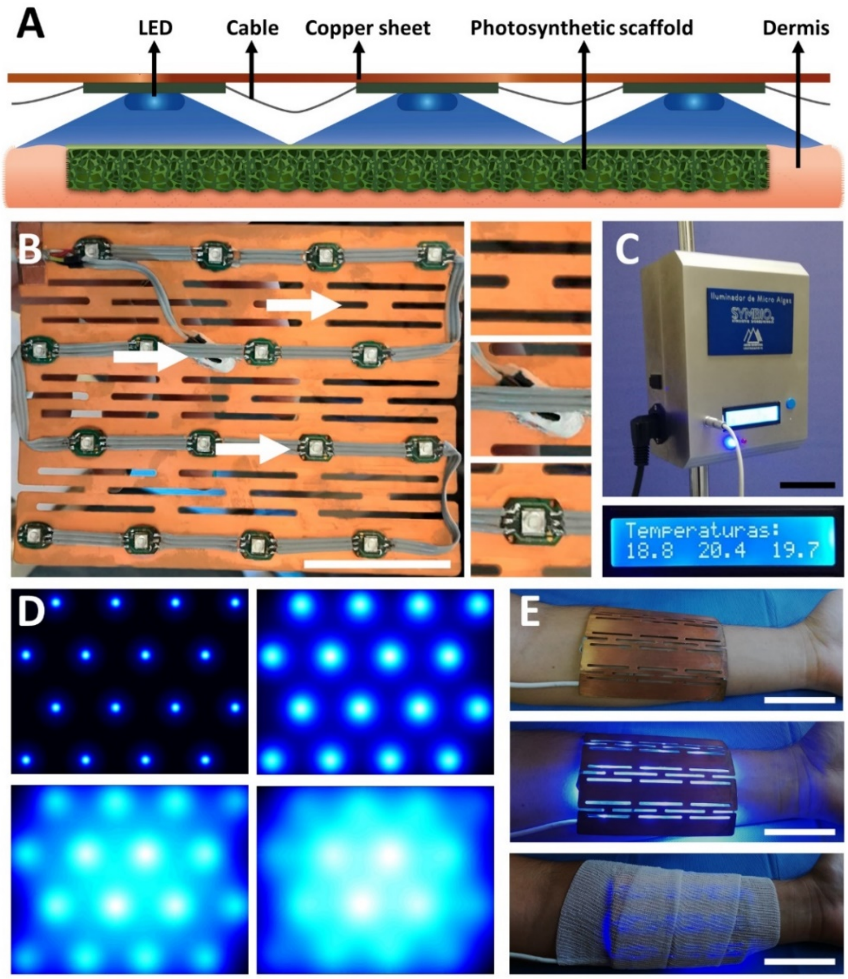

Supplementary Figure 1: Illumination device for photosynthetic scaffolds. (A) Schematic view of the illumination device placed over the photosynthetic scaffold. **(B)** Representative picture of the LED device. Arrows indicate magnified areas showing: living hinge pattern printed on the copper substrate to allow flexibility (upper), a temperature sensor (middle) and LED lights supported by a PCB and connected through flat ribbon cables (lower). **(C)** Control unit placed on a clinical holder for easy and secure handling (upper). This unit includes an LCD screen that serves as a user interface, displaying values for light intensity, battery state, and temperatures for the electronics driver, copper substrate, and battery circuit (lower). **(D)** LED intensity is adjusted through the control unit using pulse width modulation, allowing remote control of the LED illuminator. Light intensities correspond to 2, 4, 10 and 15% of the system's maximal power. **(E)** Before its clinical use, the safety of the device was evaluated on a healthy volunteer's arm. Scale bar represents 4 cm (B), 10 cm (C) and 5 cm (E).

Supplementary Table 1: Photosynthetic scaffold quality control. The absence of different microorganisms was studied and confirmed by microbiology testing at Red de Salud UC Christus, Santiago, Chile.

Microorganism	Method
**Aerobic bacteria**	Seeding on Trypcase Soy agar 5% sheep blood (bioMerieux), chocolate agar PolyViteX VCAT3 (bioMerieux) and MacConkey agar plates (BD)<br>Inoculation in Brain-Heart infusion broth (BD)
**Anaerobic bacteria**	Seeding on Schaedler agar (BD)<br>Inoculation in Thioglycollate broth
**Fungi**	Seeding on Sabouraud agar plates
**Mycobacteria**	Inoculation in BACTEC™ MGIT™ mycobacteria growth indicator tubes

Supplementary Table 2: Patient inclusion and exclusion criteria. All patients involved in the study were selected according to the following criteria.

	Specification
**Inclusion criteria**	(1) Age between 18 and 65 years<br>(2) Full thickness skin wounds<br>(3) Confirmation of wound sterility by microbiology testing<br>(4) Previously signed informed consent form
**Exclusion criteria**	(1) The following comorbidities: hypertension, diabetes mellitus, chronic liver damage, autoimmune diseases, neoplasia, immunosuppression, coronary heart disease, occlusive arterial disease, chronic smoking, drug and alcohol abuse<br>(2) Psychiatric disorders<br>(3) Injuries in the face and/or neck

Supplementary Table 3: Scaffold coverage for each patient. The use of Tegaderm® (3M) and sterile gauze as wound dressing or PDMS and negative wound pressure therapy (NPWT), as well as the need of split skin autograft is indicated for each patient.

Patient	Wound dressing	Split skin autograft
**P1**	Tegaderm® and sterile gauze	+
**P2**	Tegaderm® and sterile gauze	+
**P3**	Tegaderm® and sterile gauze	+
**P4**	Tegaderm® and sterile gauze	-
**P5**	Tegaderm® and sterile gauze	-
**P6**	PDMS membrane secured with NPWT	+
**P7**	PDMS membrane secured with NPWT	+
**P8**	PDMS membrane secured with NPWT	+
